# Anthropometric and Physical Performance of Youth Handball Players: The Role of the Relative Age

**DOI:** 10.3390/sports6020047

**Published:** 2018-05-23

**Authors:** Alba Camacho-Cardenosa, Marta Camacho-Cardenosa, Adrián González-Custodio, Ismael Martínez-Guardado, Rafael Timón, Guillermo Olcina, Javier Brazo-Sayavera

**Affiliations:** 1 Faculty of Sport Science, University of Extremadura, 10004 Cáceres, Spain; albacc@unex.es (A.C.-C); adri13agc@gmail.com (A.G.-C.); imartinezg@unex.es (I.M.-G.); rtimon@unex.es (R.T.); golcina@unex.es (G.O.); 2 Instituto Superior de Educación Física, Universidad de la República, Rivera 40000, Uruguay; jbsayavera@cur.edu.uy; 3 Laboratorio de Análisis del Rendimiento Humano, Centro Universitario de Rivera, Rivera 40000, Uruguay

**Keywords:** birth date distribution, team sport, youth sports, development, dropout

## Abstract

Background: The relative age effect is essential throughout all of the talent selection processes in sports, especially during adolescence, which leaves fewer athletes within each cohort that are born late in the selected year. The aim of the present study was to examine the role of relative age in anthropometric and physical performance characteristics of youth handball players by gender. Methods: The sample that was selected included 47 participants (male n = 23, female n = 24). The data collection included anthropometric, body compositions parameters, and physical performance levels. Results: There was a significantly higher representation of players in the first semester in comparison with the second semester, for all of the gender groups, except for the selected male players. In males, statistically significant differences were found in height, sitting height, weight, wingspan, arm and leg circumferences, and in throws speed (in support and in suspension) between those players that were born in the first and second semester. Conclusion: The results confirmed an effect of relative age in the players born in 2002 that were selected to participate in the Spanish Championship, which was different for males and females. In spite of this effect, which only appeared in females, significant differences in the anthropometric and physical conditions appeared in the male players.

## 1. Introduction

Over recent years, talent identification has produced great interest among coaches and sport scientists. The concept of ‘talent’ involves both a genetic predisposition and a capacity to improve performance through intensive practice [1]. Different models or approaches have been assumed so as to find the best way to carry out this process. These proposals included variables such as the anthropometric, physiological, psychological, and motor skill characteristics of the individual sports [2,3]. However, in many cases, the physical abilities tests that were used, did not necessarily reflect the specific needs of the team games [2]. Identifying talent in team sports at an early age is a complex process, because of the lack of precise and objective measures of performance [4].

Anthropometric parameters and physical and motor test have been identified as fundamental in order to determine the success of the performance in handball. Thus, some of the previous studies have provided the specific performance measures that could be the most useful [5]. Regarding anthropometry, some studies have demonstrated that body composition could have an influence in the game’s performance, namely: a higher hand size or a handgrip strength involves a higher and greater control of the ball, and a higher wingspan involves a higher occupation of spaces in defensive and offensive actions [6]. Granados et al. [7] showed that the higher values of fat-free mass involved a higher performance, especially because of the increase in the muscular power and strength. On the other hand, some of the studies have used physical condition parameters in order to identify talents. Srhoj et al. [8] evaluated different basic motor skills as decisive performance factors, obtaining that the fine motor skills in the upper limbs could be essential for the performance.

The relative age effect (RAE) is essential throughout the talent selection processes in team sports, such as ice hockey, football, and handball [9,10], especially in male sports during adolescence. This leaves fewer athletes within each cohort who are born late in the selected year. RAE refers to the advantage (both performance and selection) of being born early, after a cut-off date, in an annual group [11], which is the usual way to organize sport activities [12]. The relatively older players are born nearly one year before the youngest players in a cohort, and are more mature, stronger, and faster than the youngest players [13]. Consequently, these players received more attention, better training facilities, and more training time [14]. The German National Talent Selection Program belonging to the German Handball Federation, considers that, in the early stage, the less developed players must have the opportunity of being selected as players and therefore to achieve the same level of the most developed players [11]. In this sense, the maturity status and sport organization can negatively impact the drop out and talent [15]. As such, some strategies have been developed for decreasing the RAE in the selection process, such as house league [16], age-ordered shirt numbering [17], selection procedures in stages [18,19], changing selection periods [20], or introducing quotas that are related to age based on maturation or weight [21]. 

While the RAE is often large at the younger levels of sports, the effect is smaller among adults, and some researchers have even reported a possible inverse RAE [10]. In team sports, the relatively younger players that could be the most award-winning and the most valuable players [22], endure a longer career [23] or earn significantly higher wages compared with the relatively older players [24]. This is possibly because of the development of superior skills that helps them to persist in an unfavorable system [25]. In this way, based on previous arguments, there might be a need to account for the considerations of Lovell et al. [26] regarding the motor development and maturation state of players. Thus, the aim of the present study was to examine the role of relative age in the anthropometric and physical performance characteristics of the youth handball players based on gender.

## 2. Materials and Methods

### 2.1. Sample

All of the selected players (age: 13.26 ± 0.44 years; weight: 57.32 ± 8.28 kg; height: 167.42 ± 6.58 cm; 20.37 ± 2.53 kg/m^2^; and VO_2_max: 49.21 ± 3.18 mL·min·kg) that took part in the study were born in the first constituent year. Therefore, it was assumed that the selected players were to be divided by their semesters of birth. The sample included 47 (male n = 23 and female n = 24) handball players. All of the subjects that took part in the study were field-players. The goalkeepers were evaluated but their data was excluded for this analysis because of the specific required abilities [27]. The subjects had at least two years of competitive experience and trained at least twice a week. Each participant received an oral and written explanation of the procedures and their tutors provided a written informed consent. The study received the approval from the university’s ethics committee and was conducted in accordance with the Declaration of Helsinki. 

### 2.2. Procedures

The data collection took place during the National Sports Talent Program of the Spanish Handball Federation in March of 2016, with 14 year old female and male players. Coaches from the territorial handball federation selected the male and female players within the technical criteria. The data of the ‘general players’ was obtained via call or e-mail through the regional federations. 

### 2.3. Anthropometric Parameters

Firstly, the height (0.1 cm, SECA 769, seca gmbh & co.kg, Hamburg, Germany), sitting height (0.1 cm, SECA 769, seca gmbh & co.kg, Hamburg, Germany), body mass (0.1 kg, SECA 769, seca gmbh & co.kg, Hamburg, Germany), body mass index (BMI = body mass/height^2^, kg·m^−2^), arm span (0.1 cm), hand span (0.1 mm), biacromial span (0.1 cm), arm perimeter (0.1 cm), leg perimeter (0.1 cm), body fat-free mass, and body fat mass were measured through the recommended standardized techniques. The arm span, biacromial span, arm relaxed perimeter, and leg perimeter were assessed using a tape [28]. The hand span was measured (SATA, AMEFDA, Spain) as the distance between the second and the fifth finger of the dominant side [29]. The skinfold thickness at six sites (biceps, triceps, subscapular, suprailiac, abdominal, and thigh) were measured using a calliper (Slim Guide, AMEFDA, Seville, Spain) and the percentage of the fat-free mass and body fat mass were estimated using the formula from the body density [30].

### 2.4. Performance Test

Agility test: The Barrow Test [31] was carried out in order to assess the speed-agility in seconds (s), using digital chronometer (TQC DI0076, TQC B.V., Surbiton, UK).

Jumps tests: Horizontal and vertical jumps were performed in order to measure the lower limbs’ power. For the countermovement jump (CMJ) and abalakov jump (ABK), a contact platform was used (ChronoJump, Boscosystem ®, Barcelona, Spain). Two maximal jumps of each type were recorded, with 30 s rest between them. The attempt in which the highest jump length or height was obtained was taken for further analysis.

Throwing tests: The throwing velocity (m·s^−1^) was assessed using a speed gun (Sport Radar Stalker Solo 2, Stalker Radar, Richardson, Texas, USA) held 1 m to the side of the goal post, and perpendicular to the player. The players completed a maximal jump shot with 3-step run-up from 9 m. In addition, a medicine ball throw was carried out where subject’s knee had to be parallel. The weight of the ball was 3 kg. The distance (m) was measured from the front of the knee to where the ball had landed. In both of the tests, the participants threw the ball twice as far as possible, and the furthest attempt was used for the analysis.

Aerobic capacity tests: The ‘Course Navette’ test was used to assess player’s aerobic capacity. The test consisted of 20 m shuttle runs performed at increasing velocities. The highest velocity that was covered during the tests was considered as the testing score and was used to calculate the maximal oxygen uptake (VO_2_max) in mL·kg·min^−1^ [32].

### 2.5. Statistical Analyses

The data were analyzed following the standard procedures, using the statistical package for the Social Science (SPSS) v.20 for MAC (IBM, New York, NY, USA). Firstly, the normality and homoscedasticity were explored. To study the relative age effect, a Chi-Square test was performed. The absolute and relative data were reported. To analyze the differences in the anthropometric and physical performance characteristics, a t-test was carried out for the impaired samples. The mean and a 95% coefficient interval (95% CI) were reported. The level of error was set at 5%, considering the differences with a *p* ≤ 0.05.

## 3. Results

**Figure 1:**
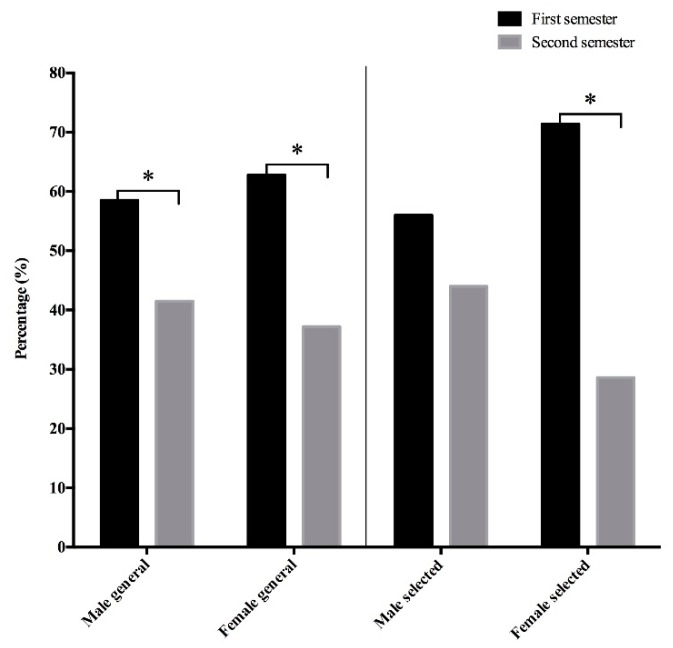
Relative age distribution by gender of selected players and the corresponding general players. * Significant difference at *p* < 0.05.

Figure 1 shows the distribution of the birth dates (semester of birth) from the players that took part in the national championship and also from those who were assessed in the present study. It was observed that there was a higher representation of players in the first half of the birth semester (H1) in comparison to second half of the birth semester (H2), which was statistically significant for all of the gender groups, except for the selected male players. However, Table 1 shows the expected frequency in the selected players following the distribution in Figure 1, where it was observed that there were only more female players than expected when compared with the distribution of the general players from Figure 1. However no statistically significant differences were found.

Table 2 and Table 3 present the data of the anthropometric characteristics from the male and female selected players, respectively. In the male group, statistically significant differences were found in the height, sitting height, weight, wingspan, and in the arm and leg circumferences between those players that were born in the first and second semester. The female players did not present statistical differences between those players that were born in the first and second semester.

Table 4 and Table 5 show the data of the physical performance of the selected male and female players, respectively. These data reported a statistically significant difference in throws speed (in support and in suspension) in the male players. However, the female players did not present significant differences between the semesters of birth.

## 4. Discussion

It is essential to take into account the RAE in the talent identification processes in handball [9], especially in the practice of the sport during adolescence. The aim of the present study was to examine the role of the relative age in the anthropometric and physical performance characteristics of the youth handball players from both genders. General male players were distributed unequally, with a higher representation of players being born in the first semester. However, the selected male players were distributed equally, and statistically significant differences were found in the anthropometric and physical conditioning between players that were born in the first and second semester. This result could be because of the fact that, in handball, there are differences in the anthropometric and physical conditioning between the players regarding their playing positions [33]. Similarly, basketball players’ body measurements are essential for assigning playing positions [34,35]. In this way, the professional players could have a moderate association between the position and arm span [35]. Therefore, even though the general selected players presented an unequal distribution, the players that were selected for these teams could have been equally distributed, because of the necessity of these different capacities.

In addition, the general female players were distributed unequally with a higher representation of players born in the first semester. In this case, the selected female players presented an unequal distribution, with more players born early in the year. There were not significant differences in anthropometric and physical conditioning between selected female players born in the both semesters. The maturation process in the female athletes finished before the male athletes, when compared [36], and the handball demands for female are different than those for males. These could therefore be the reasons for this result [37].

The anthropometric dimensions used to be the selection criteria that was used in order to select players among the youth handball players [38]. In the present study, the male players that were born in the first semester showed significantly higher values in the anthropometric and throwing test with the handball ball. Thus, these criteria could have influenced the selection procedure. In the talent selection, the anthropometry and physical condition are considered essential factors [39,40,41].

However, these could be affected by the maturity state and not take into account the other important performance factors, such as the cognitive ability or emotional competence [42]. Biological maturation on the selection process is complex and has several risks. Following puberty, the late maturing boys can catch up or even surpass their early maturing counterparts in anthropometric characteristics and physical performance [43]. Thus, coaches in youth handball teams should be aware of the maturity-selection that is based on the mentioned criteria that is not recommended as a long-term development of performance [33].

Regarding the birth distribution that has been observed in the present study, studies on RAE in handball are rare and there are plenty that show a RAE in handball sport [10,44,45,46,47,48]. In high-contact sports where the physical attributes are the determinants for success, significant RAE could be observed [14]. In addition, previous studies have shown that the RAE appeared particularly during adolescence, when the differences in physical attributes are higher [47]. This phenomenon tends to decrease with the age, probably because of the higher importance of experience and technique over physical abilities [49]. In the present study, there was an RAE in the general population of players that were selected to participate in the national championship. However, there was a lack of RAE in the selected male players. These results were similar to those found in a current study, where there was not significant RAE in the male players of a regional team [46]. Similarly, the level of performance or the short selected sample could have explained the results that were obtained [46]. Previous studies in different disciplines, which regarded the RAE with the anthropometric and performance characteristics, indicated the importance of including the assessments of these variables so as to reduce the consequences of RAE [1,2,9,10,33,41].

The female players seemed to be less likely than the male players to show RAE. Thus, as previous studies showed [9], the effect of RAE could be different between males and females. The lower competition for a place on team and the early development might have explained the differences according to gender [45]. However, the present study found significant RAE in the selected female players. However, there were no significant differences in any of the analyzed anthropometric and fitness factors. The maturity process, which is different between men and women [50,51], could be more advanced in the female gender [52].

The present study contains some limitations. It shows data for the general players from the championship who had already been selected. Thus, there was a need to compare the general distribution of players from 2002 (14 years) that played handball in Spain. Secondly, previous studies had shown a direct correlation between the RAE and the specific position in the court [10,47,53]. In this way, positions such as the left back or center, where the anthropometric characteristics are more demanding, most of the players were born in the first semester [48]. In the present study, the position in the court was not registered, and some different funding, according the anthropometric and throwing velocity between first and second semester, could have been understood, namely, the players that were born in the first semester might occupy different positions than the players that were born in the second semester, with different anthropometric values. Thus, information about position in the court is a limitation, which could be of help to partially understand the results that were obtained in the present study.

## 5. Conclusions

The results confirm an effect of the relative age in the players that were born in 2002 and that were selected to participate in the Spanish Championship that was celebrated in 2016, were different in males compared with females. Regarding the selected sample, in spite of this effect that only appeared in females, significant differences in anthropometric and physical conditions appeared in male players. Education about the existence and impact of RAE should be taken into account by those who are responsible for selecting players, by delaying competition until later and de-emphasising the ‘cultural need’ to be the best at the developmental stages. Additionally, they should design and use strategies that can reduce the consequences of this phenomenon.

## Figures and Tables

**Table 1:** Relative age distribution of selected players compared to corresponding unselected players.

		Observed Frequency						
		H1		H2				
Group	Gender	N	∆	N	∆	Total	χ^2^	*p*-Value
Selected players	M	14	−0.6	11	0.6	25	0.064	0.800
	F	20	2.4	8	−2.4	28	0.892	0.345

N total—total number of participants; H—half of birth; ∆—difference between the observed distribution and the theoretical expected distribution.

**Table 2:** Anthropometric characteristics in male players divided by birth semester.

Variable	H1 (n = 14)	H2 (n = 11)	∆%	*d*	*p*-Value
Height, cm	175.14 (171.43, 178.86)	168.18 (161.82, 174.54)	−3.97%	0.99	0.039
Sitting height, cm	87.11 (85.12, 89.09)	83.14 (79.91, 86.37)	−4.56%	0.16	0.024
Weight, kg	64.26 (58.19, 70.34)	53.56 (47.22, 59.91)	−16.65%	0.72	0.015
BMI, kg/m^2^	20.87 (19.36, 22.37)	18.85 (17.33, 20.37)	−9.68%	0.42	0.054
Wingspan, cm	180.21 (176.28, 184.15)	170.82 (163.42, 178.21)	−5.21%	0.48	0.015
Hand span, mm	76.05 (70.37, 81.37)	79.33 (76.08, 82.59)	4.31%	10.31	0.297
Biacromial span, cm	35.71 (34.37, 37.06)	34.50 (33.03, 35.97)	−3.39%	1.05	0.197
Arm circumference, cm	23.90 (22.45, 25.35)	21.65 (20.42, 22.88)	−9.41%	0.08	0.020
Leg circumference, cm	51.41 (48.94, 53.88)	46.62 (42.99, 50.24)	−9.32%	0.05	0.021
Skinfold suprailiac, mm	10.45 (6.67, 14.24)	6.76 (4.32, 9.19)	−35.31%	1.40	0.107
Abdominal skinfold, mm	20.17 (10.79, 29.54)	10.70 (6.91, 14.48)	−46.95%	1.47	0.078
Biceps skinfold, mm	6.50 (3.76, 9.24)	4.06 (2.61, 5.51)	−37.54%	1.01	0.129
Triceps skinfold, mm	11.07 (7.54, 14.60)	8.24 (6.16, 10.32)	−25.56%	1.78	0.176
Subscapular skinfold, mm	9.88 (7.14, 12.62)	7.60 (5.84, 9.37)	−23.08%	1.40	0.167
Thing skinfold, mm	10.80 (8.11, 13.51)	7.88 (6.11, 9.64)	−27.04%	0.74	0.076
Fat mass, %	10.32 (7.98, 12.67)	8.02 (6.80, 9.26)	−22.29%	0.65	0.096

Data are expressed as mean (95% CI). H—half of birth; ∆%—percentage difference between birth semesters; *d—*Cohen’s effect size.

**Table 3:** Anthropometric characteristics in female players divided by birth semester.

Variable	H1 (n = 20)	H2 (n = 8)	∆%	*d*	*p*-Value
Height, cm	162.25 (159.43, 165.07)	165.50 (159.85, 171.15)	2.00%	9.65	0.224
Sitting height, cm	84.91 (83.02, 86.80)	83.50 (80.94, 86.06)	−1.66%	2.14	0.383
Weight, kg	55.67 (51.88, 59.45)	52.24 (43.96, 60.52)	−6.16%	5.56	0.350
BMI, kg/m^2^	21.13 (19.88, 22.37)	18.98 (16.79, 21.17)	−10.18%	0.49	0.064
Wingspan, cm	161.25 (157.35, 165.15)	166.38 (160.72, 172.03)	3.18%	12.68	0.135
Hand span, mm	71.16 (68.76, 73.55)	71.45 (68.54, 74.36)	0.41%	4.59	0.884
Biacromial span, cm	34.85 (33.50, 36.20)	35.00 (32.65, 37.35)	0.43%	3.00	0.901
Arm circumference, cm	22.72 (21.79, 23.66)	21.38 (19.24, 23.51)	−5.90%	0.93	0.148
Leg circumference, cm	51.43 (49.18, 53.68)	46.94 (41.63, 52.26)	−8.73%	1.09	0.052
Skinfold suprailiac, mm	11.80 (9.75, 13.85)	9.34 (3.85, 14.82)	−20.85%	2.91	0.255
Abdominal skinfold, mm	23.42 (19.27, 27.56)	20.71 (12.61, 28.81)	−11.57%	6.57	0.483
Biceps skinfold, mm	9.42 (7.98, 10.85)	8.67 (4.86, 12.47)	−7.96%	3.06	0.615
Triceps skinfold, mm	15.85 (13.88, 17.82)	13.92 (9.57, 18.27)	−12.18%	2.78	0.314
Subscapular skinfold, mm	12.32 (10.81, 13.82)	10.96 (7.05, 14.87)	−11.04%	2.59	0.385
Thing skinfold, mm	16.10 (14.27, 17.93)	17.79 (10.30, 25.28)	10.50%	8.12	0.486
Fat mass, %	17.27 (15.76, 18.79)	16.20 (11.83, 20.56)	−6.20%	3.16	0.512

Data are expressed as mean (95% CI). H—half of birth; ∆%—percentage difference between birth semesters; *d—*Cohen’s effect size.

**Table 4:** Physical performance characteristics in male players divided by birth semester.

Variable	H1 (n = 14)	H2 (n = 11)	∆%	*d*	*p*-Value
Long jump, m	1.93 (1.80, 2.06)	1.82 (1.75, 1.89)	−5.70%	0.05	0.165
Abalakov, cm	32.22 (28.07, 36.36)	30.70 (27.95, 33.43)	−4.72%	4.11	0.536
TMBK, m	4.05 (3.79, 4.32)	3.74 (3.55, 3.93)	−7.65%	0.06	0.065
SUTB, km/h	81.14 (77.76, 84.52)	70.36 (65.86, 74.86)	−13.29%	4.50	<0.001
STTB, km/h	74.93 (71.49, 78.37)	68.82 (64.91, 72.72)	−8.15%	0.23	0.017
Speed-agility, s	10.30 (10.05, 10.54)	10.24 (9.95, 10.54)	−0.58%	0.37	0.760
VO_2_max, mL·min·kg	49.69 (47.80, 51.57)	51.86 (49.57, 54.14)	4.37%	5.51	0.119

Data are expressed as mean (95% CI). H—half of birth; ∆%—percentage difference between birth semesters; *d—*Cohen’s effect size; TMBK—throwing medicine ball on knees; SUTB—suspension throwing ball; STTB—standing throwing ball.

**Table 5:** Physical performance characteristics in female players divided by birth semester.

Variable	H1 (n = 20)	H2 (n = 8)	∆%	*d*	*p*-Value
Long jump, m	1.61 (1.51, 1.70)	1.62 (1.52, 1.71)	0.62%	0.17	0.906
Abalakov, cm	29.38 (27.29, 31.48)	29.00 (25.78, 32.22)	−1.29%	3.78	0.834
TMBK, m	3.32 (3.10, 3.54)	3.02 (2.90, 3.14)	−9.04%	0.01	0.092
SUTB, km/h	62.95 (60.60, 65.30)	61.00 (57.45, 64.55)	−3.10%	2.68	0.343
STTB, km/h	62.25 (59.98, 64.52)	60.63 (56.71, 64.54)	−2.60%	3.15	0.426
Speed-agility, s	10.73 (10.50, 10.96)	10.83 (10.46, 11.20)	0.93%	0.57	0.615
VO_2_max, mL·min·kg	47.36 (45.36, 49.36)	47.37 (43.50, 51.23)	0.02%	4.46	0.999

Data are expressed as mean (95% CI). H—half of birth; ∆%—percentage difference between birth semesters; *d—*Cohen’s effect size; TMBK—throwing medicine ball on knees; SUTB—suspension throwing ball; STTB—standing throwing ball.
